# Enhanced Liver Fibrosis Score: Is It Useful for Evaluation of Fibrosis Severity in Chronic Hepatitis C Infection?

**DOI:** 10.7759/cureus.21168

**Published:** 2022-01-12

**Authors:** Manish Kumar, Roshan George, Venkatesh Vaithiyam, Puja Sakhuja, Amol S Dahale, Aman Dayal, Ashok Dalal, Ujjwal Sonika, Sanjeev Sachdeva, Ajay Kumar

**Affiliations:** 1 Department of Gastroenterology, Govind Ballabh Pant Institute of Postgraduate Medical Education and Research, New Delhi, IND; 2 Department of Pathology, Govind Ballabh Pant Institute of Postgraduate Medical Education and Research, New Delhi, IND; 3 Department of Statistics, Maulana Azad Medical College, New Delhi, IND

**Keywords:** elf, transient elastography, metavir, hcv, liver biopsy

## Abstract

Introduction: The assessment of liver fibrosis is important in patients with chronic hepatitis C (CHC). In recent years, non-invasive tests like enhanced liver fibrosis (ELF) have been developed as an alternative to liver biopsy for estimating the severity of liver fibrosis. Therefore, we aimed to assess whether the ELF score can be used for fibrosis severity estimation using liver biopsy as the gold standard.

Materials and methods: Forty-nine patients with CHC were enrolled in this study. Liver biopsy, ELF assessment, and transient elastography (TE) were performed in all patients, and severity of fibrosis on histopathology was assessed by meta-analysis of histological data in viral hepatitis (METAVIR) score. In addition, the diagnostic performance of ELF was evaluated by receiver operator characteristic curve (ROC) analyses, and liver biopsy histopathology was taken as the gold standard for the severity of liver fibrosis.

Results: The area under receiver operator characteristic curve (AUROC) for significant fibrosis of ELF score was 0.64 (95% confidence interval {CI}, 0.48-0.79) and of TE was 0.85 (95% CI, 0.73-0.96). The AUROC for advance fibrosis of ELF was 0.77 (95% CI, 0.57-0.97) and TE was 0.98 (95% CI, 0.94-1.0). The calculated cut-offs of ELF overestimated fibrosis in 53.06% (26/49) of patients and underestimated fibrosis in 6.12% (3/49) patients. AUROC of TE was significantly better than ELF for diagnosis of significant fibrosis (p=0.004) and advanced fibrosis (p=0.034).

Conclusion: The ELF score can be used for estimating the severity of fibrosis but it is inferior to TE in estimating liver fibrosis severity.

## Introduction

Hepatitis C virus (HCV) is one of the leading causes of chronic liver disease globally, and it is estimated that 170-185 million people have chronic HCV infection [1]. The assessment of liver fibrosis is important in patients with HCV infection. Patients with advanced fibrosis or cirrhosis need to be followed despite achieving sustained virological response (SVR). In addition, a longer duration of therapy is needed in patients with HCV-related cirrhosis having infection by genotype 3. Liver biopsy is still considered the gold standard method for assessing fibrosis severity in chronic liver diseases. Still, it is invasive and has a small but definite risk of mortality [2]. In addition, sampling error and significant intra- and inter-observer variability in histological staging affect the accuracy of liver biopsy in the estimation of fibrosis severity [3,4].

In recent years, non-invasive tests have been developed as a substitute to liver biopsy for estimating the severity of liver fibrosis. The non-invasive tests available are biomarkers, indirect and direct biomarkers, and ultrasound or magnetic resonance-based tests. The commercially available biomarkers tests are Fibrometer, Forn’s Index, FibroTest, Hepascore, FIB-4, and enhanced liver fibrosis (ELF) score [5].

The ELF score is estimated from an algorithm that uses three fibrosis markers - amino-terminal peptide of type III procollagen (PIIINP), hyaluronic acid (HA), and tissue inhibitor of matrix metalloproteinases-1 (TIMP-1) [6]. The strengths of the ELF score are high reproducibility, non-invasive, and good diagnostic performance in the assessment of liver fibrosis [7].

Liver stiffness measurement (LSM) is useful in estimating liver fibrosis, and the most widely used method is transient elastography (TE) (FibroScan; Paris, France: Echosens) [8]. The TE already has been validated in a large cohort of HCV patients as a non-invasive test for liver fibrosis severity [9]. However, the major drawbacks of TE are the low sensitivity in the intermediate stage of fibrosis, technical failure in about 3% of cases, and unreliable results in an additional 16% of cases [10]. Also, edema, space-occupying lesions, inflammation, cholestasis, and congestion interfere in measuring the LSM by TE.

Previously, biomarkers have been compared with TE in the estimation of the severity of fibrosis in HCV infection; however, in most studies, liver biopsy was not performed, which is still considered the gold standard method for estimation of liver fibrosis severity [11]. Therefore, the present study aimed to determine whether the ELF score can also be used for fibrosis severity estimation and compare its diagnostic performance with TE in fibrosis severity estimation using liver biopsy as the gold standard.

## Materials and methods

Patients population

Patients with chronic hepatitis C (HCV RNA positive by real-time polymerase chain reaction, the lower limit of detection 10 IU/ml) aged 18-60 years, attended at Department of Gastroenterology, GIPMER, Delhi, India, between January 2017 and June 2018, were evaluated for enrollment for study. Patients previously treated or on treatment for HCV, decompensated liver disease, hepatocellular carcinoma (HCC), extrahepatic malignancy, pregnancy, alcohol ingestion more than 40 g/day in males and 20 g/day in females, co-infection with human immunodeficiency virus and or hepatitis B virus, co-morbidities (chronic kidney disease, congestive heart failure), inadequate liver biopsy specimen, failure to measure valid TE reading, and body mass index > 30 kg/m^2^ were excluded from the study.

We took written informed consent from all subjects. The study was approved by the institutional ethics committee in Maulana Azad Medical College and associated Lok Nayak, Govind Ballabh Pant Hospital, Guru Nanak Eye Centre, New Delhi via letter numbered F.No./11/IEC/MAMC/2016 and was following the Helsinki declaration.

Liver histopathology

The liver biopsy was performed using the true-cut liver biopsy needles (Bard biopsy gun {Franklin Lakes, NJ: CR Bard Inc}, 16-gauge by 22-mm penetration depth; 17-mm sample notch) under the fluoroscopic guidance from the right mid-axillary lower-intercostal approach (10th or 11th intercostal space). The biopsy needle was not be passed more than three times, and the number of needle passes per biopsy was decided by the liver biopsy size obtained. After the liver biopsy, the patients were kept under observation for the next six hours, during which blood pressure and heart rate were monitored every 15 minutes for two hours and then every 30 minutes for the next four hours. After that patients were admitted in case of complications and further complications were managed as per established standard protocol. The liver biopsy specimen was cut into six consecutive sections (i.e., levels) and 4 μm thick from paraffin-embedded tissue, stained with hematoxylin and eosin, Masson’s trichrome, and orcein. The liver biopsy sample was considered adequate if 10 complete portal triads are seen in the biopsy specimen. The severity of fibrosis in liver biopsy was assessed by meta-analysis of histological data in viral hepatitis (METAVIR) fibrosis scoring system (F0: no fibrosis, F1: fibrosis in portal tract without septa, F2: portal fibrosis with rare septa, F3: numerous septa without cirrhosis, and F4: cirrhosis) [12]. The fibrosis severity on liver biopsy specimen was taken as the significant fibrosis if METAVIR score was ≥ F2 and advanced fibrosis if METAVIR score was ≥ F3. The liver biopsy was evaluated by a single expert pathologist who was blinded to TE and ELF values.

Enhanced liver fibrosis score and transient elastography

The TE estimation and blood sample collection for ELF score were done in fasting state on the same day. For ELF, 5 ml of the fresh blood sample was collected and sent to a laboratory immediately for storage and further processing. The quantitative estimation of HA, PIIINP, and TIMP-1 was done on serum obtained from blood; the estimation was done by chemiluminescent microparticle immunoassay. To calculate the ELF score, the ADVIA Centaur system (Erlangen, Germany: Siemens Healthineers) was used. To calculate the HA, PIIINP, and TIMP-1 assays, values obtained from the ADVIA Centaur were used by the following equation/algorithm which was used to calculate the ELF score (concentrations {C} of each assay are in ng/ml).

ADVIA Centaur CP: ELF score = 2.494 + 0.846 ln (CHA) + 0.735 ln (CPIIINP) + 0.391 ln (CTIMP-1)

The TE was measured by the trained operator using an M probe. As per previous guidelines, TE results obtained from 10 measurements with a success rate above 60% and an interquartile range of ≤ 30% were taken as valid results.

Statistical analysis

Statistical analyses were performed using the SPSS version 18 (Armonk, NY: IBM Corp.). The continuous variables are expressed as mean with SD and categorical variables as numbers and percentages. The ELF score optimal cut-off values for no or mild fibrosis (F0-F1), significant fibrosis (≥ F2), and advanced fibrosis/cirrhosis (≥ F3) were calculated with sensitivity, specificity, and diagnostic accuracy of cut-off values. For, the ELF and TE scores diagnostic performance in predicting significant fibrosis (≥ F2) and advanced fibrosis (≥ F3) was done by estimating the areas under the receiver operator characteristic curve (AUROC). Comparison of AUCs was performed according to the DeLong method. The probability level of p < 0.05 was set for statistical significance. The sample size of 50 for the study was decided as invasive liver biopsy used as the gold standard in the present study.

## Results

A total of 122 patients were evaluated during the study period, of which 73 patients were excluded (decompensated liver disease n=22, HBV co-infection n=8, alcohol abuse n=12, previously treated for HCV n=11, didn’t give consent n=8, BMI > 30 kg/m^2^=8 patients, HCC n=3, and outliner ELF value n=1) and remaining 49 patients were included. The mean length of the liver biopsies specimen was 23.0±1.10 mm, and the mean number of portal tracts seen in liver biopsy was 11±4.0. The baseline characteristics of the study population are summarized in Table 1.

**Table 1:** Baseline characteristics of study population. AST: aspartate aminotransferase; ALT: alanine aminotransferase; SALP: serum alkaline phosphatase; HCV RNA viral load: hepatitis C virus ribonucleic acid viral load; T protein: total protein

Characteristic	Patients (n=49)
Sex (male/female)	29/20 (59.2%)
Age (years)	34.45±10.07
Body mass index (kg/m^2^)	22.50±3.50
Hemoglobin (g/dl)	13.61±1.68
Total leukocyte count (cells/mm^3^)	7382.04±2051.32
Platelet count (10^9^/l)	207.99±76.6
Blood urea (mg/dl)	24.43±6.76
Serum creatinine (mg/dl)	0.80±0.23
International normalized ratio	1.07±0.10
Bilirubin (mg/dl)	0.57±0.28
AST (U/l)	75.14±45.70
ALT (U/l)	119.67±89.20
SALP (IU/l)	106.06±36.51
T. protein (g/dl)	7.68±0.65
Serum albumin (g/dl)	4.33±0.37
HCV RNA viral load (log)	5.43±1.12
HCV genotype 1, 3	8 (16.4%), 41 (83.6%)

Fibrosis severity assessment by histopathology, ELF score, and TE

The fibrosis severity on liver biopsy was as follows: no or mild fibrosis (F0-F1) n=31, 63.3% patients; moderate fibrosis (F2) n=11, 22.4% patients; and advanced fibrosis (≥ F3) n=7, 14.3% patients. The fibrosis severity categorization on ELF was as follows: no or mild fibrosis (F0-F1) n=14, 28.6% patients; moderate fibrosis (F2) n=5, 10.2% patients; and advanced fibrosis/cirrhosis n=30, 61.2% patients. The fibrosis severity on TE was as follows: no or mild fibrosis (F0-F1) n=24, 49.0% patients; moderate fibrosis (F2) n=19, 38.8% patients; and advanced fibrosis (≥ F3) n=6, 12.2% patients.

ELF sensitivity, specificity, and diagnostic accuracy for significant fibrosis and advanced fibrosis

The ELF score cut-off of 8.85 for significant fibrosis has a sensitivity of 89.5% and specificity of 38.7%, and ELF score of 9.19 has a sensitivity of 87.5% and specificity of 42.9% for advanced fibrosis/cirrhosis (Table 2).

**Table 2:** Fibrosis categorization on liver histopathology, ELF, and TE. ELF: enhanced liver fibrosis; TE: transient elastography

Fibrosis	Liver histology n (%)	ELF n (%)	TE n (%)
Absent or mild fibrosis (F0-F1)	31 (63.3)	14 (28.6)	24 (49.0)
Moderate fibrosis (F2)	11 (22.4)	5 (10.2)	19 (38.8)
Advanced fibrosis/cirrhosis (F3-F4)	7 (14.3)	30 (61.2)	6 (12.2)
Total	49 (100)	49 (100)	49 (100)

Diagnostic accuracy of ELF and TE for significant fibrosis and advanced fibrosis

The AUROC of ELF score was 0.64 (95% confidence interval {CI}, 0.48-0.79) and of TE was 0.85 (95% confidence interval {CI}, 0.73-0.96) for significant fibrosis (≥ F2). The AUROC of ELF score was 0.77 (95% confidence interval {CI}, 0.57-0.97) and AUROC of TE was 0.80 (95% confidence interval {CI}, 0.94-1.0) for advanced fibrosis/cirrhosis (≥ F3). Comparing the AUROC of ELF score and TE, TE was significantly better for diagnosis of significant fibrosis (p=0.004) and advanced fibrosis (p=0.034). Based on the AUROC, the calculated cut-off points for ELF score for significant fibrosis and advance fibrosis/cirrhosis were 8.85 (sensitivity: 89.5%, specificity: 38.7%) and 9.19 (sensitivity: 87.5%, specificity: 42.9%), respectively (Tables 3, 4).

**Table 3:** Diagnostic performance for significant fibrosis and advanced fibrosis/cirrhosis of ELF score and TE. ELF: enhanced liver fibrosis; TE: transient elastography

Method	Significant fibrosis ( F ≥ 2)	Advanced fibrosis/cirrhosis (F ≥ 3)
ELF score	0.64 (0.48-0.79)	0.77 (0.57-0.97)
TE	0.85 (0.73-0.96)	0.98 (0.94-1.0)

**Table 4:** ELF score cut-off values for significant fibrosis and advanced fibrosis/cirrhosis. ELF: enhanced liver fibrosis

Value	Cut-off ELF score value	Sensitivity (%)	Specificity (%)	Positive predictive value (%)	Negative predictive value (%)
Significant fibrosis (F ≥ 2)	8.85	89.5	38.7	47.2	85.7
Advance fibrosis/cirrhosis (F ≥ 3)	9.19	87.5	42.9	22.5	94.7

## Discussion

The assessment of liver fibrosis is essential in patients with the liver disease since long-term liver-related morbidity and mortality depend on the severity of fibrosis. Liver biopsy is still considered the gold standard method for assessing the severity of fibrosis despite its limitations of being invasive [3,4].

The non-invasive markers are biomarkers and radiological tests which assess liver stiffness. Most of the biomarkers are available and may be used alone or in combination, such as Fibrometer, Forn’s Index, FibroTest, Hepascore, FIB-4, and enhanced liver fibrosis score (ELF) [5]. The ELF test is a logarithmic algorithm combining quantitative serum measurements of three markers of hepatic extracellular matrix metabolism - amino-terminal peptide of type III procollagen (PIIINP), tissue inhibitor of matrix metalloproteinases-1 (TIMP-1), and hyaluronic acid (HA) [6]. Two cohort studies by Martinez et al. and Petersen et al. found that 63% of patients in the cohort and 74.7% in the 112 cohorts could avoid biopsy by using ELF as surrogate fibrosis, respectively [13,14].

To the best of our knowledge, this is the first Indian study that compares the ELF score and TE with liver histology for assessments of liver fibrosis and cirrhosis in chronic hepatitis C patients. The present study evaluates the diagnostic accuracy of ELF score in chronic hepatitis C patients for fibrosis severity. Most patients had no or mild fibrosis (63.3%), or moderate fibrosis (30.6%), and only 6.1% of patients had severe fibrosis or cirrhosis in the liver biopsy. A study by Fernandes et al. identified the severity of fibrosis similar to our study. They showed that 54.2% of patients with no or mild fibrosis, 39.2% patients with moderate fibrosis, and 6.6% patients with severe fibrosis/cirrhosis [15]. Studies on the natural history of chronic hepatitis C infection have shown that the median time of development of cirrhosis after HCV infection is 30 years [16]. Our study had a younger population with a mean age of 34.45±10.07 years; thus, they might have a shorter duration of HCV infection, so most of our patients did not have any fibrosis or mild fibrosis. Other reasons can be that liver biopsy is not required once cirrhosis has been diagnosed based on clinical, endoscopy, and imaging findings. In any out-patient setting, most HCV cases at the time of presentation will be having mild to moderate fibrosis as only 20% of HCV-infected patients develop cirrhosis over 15-20 years [17].

The ELF score classified 10.2% of patients as moderate fibrosis and 61.2% as advanced fibrosis/cirrhosis. Thus, the ELF score overestimated the fibrosis in HCV patients compared to liver biopsy and TE. As per the present study, the ELF score had reasonably fair accuracy for diagnosing advanced fibrosis and low accuracy in diagnosing mild to moderate fibrosis. In comparison, TE had better diagnostic accuracy than ELF score for diagnosing all stages of fibrosis; this contrasts with the study performed by Fernandes et al. and Friedrich-Rust et al. [15,18]. The inferior performance of ELF score in detecting mild to moderate fibrosis as compared to the previous studies might be due to different genotype of HCV in our patients and higher proportions of (59%) males in our study as compared to 34% in a study performed by Fernandes et al. [15]. The previous studies have shown that males have significantly higher ELF scores than females [19].

The ELF score cut-off points overestimated the fibrosis stage; the one possible reason can be that mixed etiologies of liver disease were included in the studies to establish the cut-off points of the ELF score. To summarize, ELF score had fair diagnostic accuracy for advanced fibrosis and significant fibrosis though it was inferior to TE. However, it was not possible to suggest an ELF score for categorizing fibrosis as significant overlap was seen between fibrosis severity categories.

There are a few drawbacks to our study. First, the sample size could have been larger. Second, genotype 1 patients are less in our study. Finally, we didn’t do multivariate analysis on the effect of necroinflammation on ELF score. The strengths of our study were the inclusion of liver biopsy was used as the gold standard, the biopsy was assessed by an expert, and study conditions matching real-world scenarios.

## Conclusions

In this study, most HCV patients presented with no or mild fibrosis. The ELF score was not an excellent non-invasive fibrosis marker; the TE was a good marker for all fibrosis stages. In addition, there was an overestimation of fibrosis compared to the histological evaluation with ELF score, suggesting that new cut-off points need to be established to improve the performance of ELF score for the discrimination of different stages of fibrosis in patients with chronic hepatitis C (CHC). Finally, it is not possible to suggest a new or improved score for categorizing fibrosis from our study. In the present case, the ELF score range is significantly overlapping between all three categories of fibrosis.
